# Dopamine Modulation of *Drosophila* Ellipsoid Body Neurons, a Nod to the Mammalian Basal Ganglia

**DOI:** 10.3389/fphys.2022.849142

**Published:** 2022-04-14

**Authors:** Giovanni Frighetto, Mauro A. Zordan, Umberto Castiello, Aram Megighian, Jean-René Martin

**Affiliations:** ^1^ Department of General Psychology, University of Padova, Padova, Italy; ^2^ Institut des Neurosciences Paris-Saclay, Université Paris-Saclay, CNRS, Saclay, France; ^3^ Department of Biology, University of Padova, Padova, Italy; ^4^ Padova Neuroscience Center, University of Padova, Padova, Italy; ^5^ Department of Biomedical Sciences, University of Padova, Padova, Italy

**Keywords:** central complex, functional calcium brain imaging, dopamine 1-like receptors, action selection, nicotine response, basal ganglia

## Abstract

The central complex (CX) is a neural structure located on the midline of the insect brain that has been widely studied in the last few years. Its role in navigation and goal-oriented behaviors resembles those played by the basal ganglia in mammals. However, the neural mechanisms and the neurotransmitters involved in these processes remain unclear. Here, we exploited an *in vivo* bioluminescence Ca^2+^ imaging technique to record the activity in targeted neurons of the ellipsoid body (EB). We used different drugs to evoke excitatory Ca^2+^-responses, depending on the putative neurotransmitter released by their presynaptic inputs, while concomitant dopamine administration was employed to modulate those excitations. By using a genetic approach to knockdown the dopamine 1-like receptors, we showed that different dopamine modulatory effects are likely due to specific receptors expressed by the targeted population of neurons. Altogether, these results provide new data concerning how dopamine modulates and shapes the response of the ellipsoid body neurons. Moreover, they provide important insights regarding the similitude with mammals as far as the role played by dopamine in increasing and stabilizing the response of goal-related information.

## Introduction

The insect central complex (CX) represents an integrative structure which provides spatial representation for locomotor control (Pfeiffer and Homberg, 2014). It has been an intriguing neural structure for neuroscientists since several decades. Lately, it has become a highly debated topic where several research groups have thoroughly investigated its connectome, transcriptome and shown its functional role in navigation and goal-oriented behaviors (Seelig and Jayaraman, 2015; Green et al., 2019; Turner-Evans et al., 2020; Hulse et al., 2021).

**FIGURE 1:**
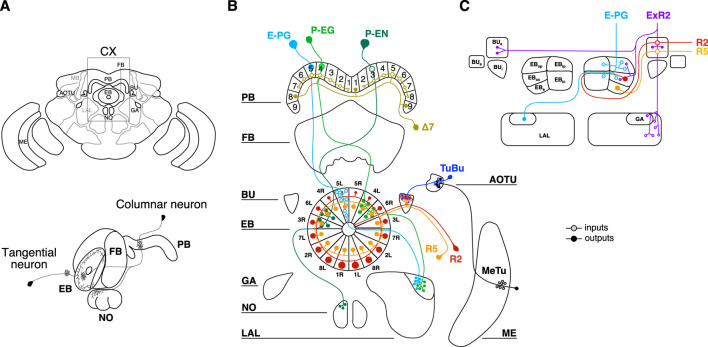
Central complex and type of neurons innervating the ellipsoid body. **(A)** On the top, cartoon of the fly brain with a window framing the central complex (CX). Four different neuropils composed the CX: protocerebral bridge (PB); fan-shaped body (FB); ellipsoid body (EB) and noduli (NO). Other brain regions represent important hubs for the information transmitted to/from the CX: medulla (ME); anterior optic tubercle (AOTU); bulb (BU); gall (GA) and lateral accessory lobe (LAL). On the bottom, example of two different types of neurons innervating the CX: tangential and columnar neurons. **(B)** Circuit diagram of the columnar neurons involved in encoding the visual inputs and in translating them to information for navigation and goal-oriented behaviors. E-PG neurons are columnar neurons receiving inputs in the EB and sending outputs to PB and GA. P-EG and P-EN are columnar neurons receiving inputs in the PB and sending outputs to the EB. Δ7 neurons receive inputs from seven glomeruli in the PB and send output to three glomeruli spaced out by seven glomeruli. R2 and R5 are tangential neurons innervating similar regions of the EB and BU. MeTu neurons, relaying visual information to the AOTU, are also depicted. **(C)** EB domains innervated by the neurons object of our study: E-PG, R2 and R5. ExR2 (also known as PPM-EB) dopaminergic neurons are also depicted (image adapted from Omoto et al., 2018).

The CX is an ensemble of four neuropils located along the midline of the protocerebrum (Figure 1A). Two main types of neurons innervate the CX (Figure 1A): tangential neurons, which form strata within one neuropil, and columnar neurons, which connect different neuropils perpendicularly (Hanesch et al., 1989). Among the four neuropils the lower division of the central body, also known as ellipsoid body (EB), constitutes the entry point of visual inputs to the CX through the anterior optic tubercle (AOTU) (Seelig and Jayaraman, 2013; Omoto et al., 2017; Hardcastle et al., 2021). Visual information is conveyed by the tubercular-bulbar neurons (TuBu) to the bulb (BU), towards which the tangential neurons of the EB project their dendrites (Shiozaki and Kazama, 2017; Sun et al., 2017). In flies, these tangential neurons send their axons to different layers of the EB forming a densely packed toroid of synapses (Figure 1B). Because of their ringlike shape, they are called ring neurons (R-neurons) and different subclasses have been defined on the basis of the layer (i.e., R1-6) and the domains innervated in the EB (Omoto et al., 2018). The columnar neurons innervating the EB arborize in different circular sectors along the coronal plane and they maintain a segregated organization projecting to the protocerebral bridge (PB) and to other compartments of the CX, such as gall (GA)—small regions on the “shoulder” of the lateral accessory lobe (LAL)—or noduli (Hanesch et al., 1989). Besides the innervation pattern, different types of columnar neurons have been classified depending on the radial morphology of their circular sectors: the wedge-shaped and the tile-shaped arborizations (Wolff et al., 2015).

The most famous wedge-shaped neurons are probably the E-PG (Figure 1B), where the first letter stands for the region receiving mainly inputs, that is the EB, and the ones after the hyphen stand for the output regions, that is the PB and the GA (Wolff and Rubin, 2018). These neurons, which segment the toroid in a way that resembles the slices of a pie (i.e., wedges), have been shown to maintain a representation of the fly’s heading direction also in the absence of direct sensory inputs through attractor-like dynamics (Seelig and Jayaraman, 2015; Green et al., 2017; Turner-Evans et al., 2017). More generally, the E-PG neurons may reflect one component of a complex interface between heading and action selection which in turn underlies complex abilities such as navigation (Green et al., 2019; Dan et al., 2021). On the other hand, tile-shaped neurons such as the P-EG instead (Figure 1B), occupy the surface volume of the caudal EB and they innervate larger circular sectors (i.e., tiles) than the wedges (Wolff et al., 2015).

Finally, another subclass of ring neurons, called ExR-neurons (extrinsic ring neurons), arborize in the EB but also outside of it (Hanesch et al., 1989). The ExR2 neurons correspond to the PPM3-EB dopaminergic neurons innervating EB, BU and the LAL (Figure 1C) (Omoto et al., 2018; Hulse et al., 2021).

Remarkably, extensive correspondences in heritable ontogeny, neuroanatomical organization and function between the vertebrate basal ganglia and the insect central complex (CX) have been put forward. Specifically, similarities have been found regarding the embryological derivation, orthologous genetic specification, neurochemicals and physiological properties (Strausfeld and Hirth, 2013a; Strausfeld and Hirth, 2013b). Furthermore, correspondences of computational mechanisms mediated by dopamine, which subtend the selection and maintenance of actions in vertebrates and insects, have been proposed (Fiore et al., 2015). In a similar manner, the dopaminergic fluctuations in the basal ganglia and the CX might mediate the switch from stable to unstable patterns of selection and vice versa through the differential effects of the direct and indirect pathways (Humphries et al., 2012; Fiore et al., 2015).

Whereas some behavioral effects of dopamine in the CX have already been shown (Lebestky et al., 2009; Kong et al., 2010; Kottler et al., 2019), no data regarding its role in the neurophysiological modulation of CX and even less supporting the existence of distinct pathways—based on different type of receptors—are available so far.

Here, we asked whether dopamine modulates EB neurons and whether different types of receptors could differentially mediate their activation levels. The aim of this study was to understand whether neural circuits involved in goal-oriented behaviors in flies respond to dopamine in a way that is similar to the mammalian basal ganglia (Green et al., 2019; Kottler et al., 2019; Dan et al., 2021). We studied the effects of dopamine on two tangential (i.e., R2 and R5) and one columnar (i.e., E-PG) neurons (Wolff et al., 2015; Omoto et al., 2018). By taking advantage of a functional *in vivo* bioluminescence Ca^2+^ imaging technique (Martin et al., 2007), we recorded the activity of specific neural populations in response to excitatory drugs with and without a previous dopamine application.

Our results show that: 1) the E-PG neurons are modulated by dopamine and two subtypes of dopamine 1-like receptors, Dop1R1 and Dop1R2, influence the state of their excitability in opposite ways; 2) two populations of R-neurons, R2 and R5,—likely expressing Dop1R1 and dopamine 2-like receptors (D2R) respectively—are modulated by dopamine, which increases the response in R2 and decreases the response in R5 neurons.

These results suggest that dopamine affects the excitability state of the EB neurons and that different pathways, involving different type of dopamine receptors—as seen in vertebrate basal ganglia—modulate their responses. We propose that dopamine might contribute to the selection of goal-directed behaviors by exciting and inhibiting different subsets of tangential and columnar neurons.

## Materials and Methods

### Fly Stocks

**FIGURE 2:**
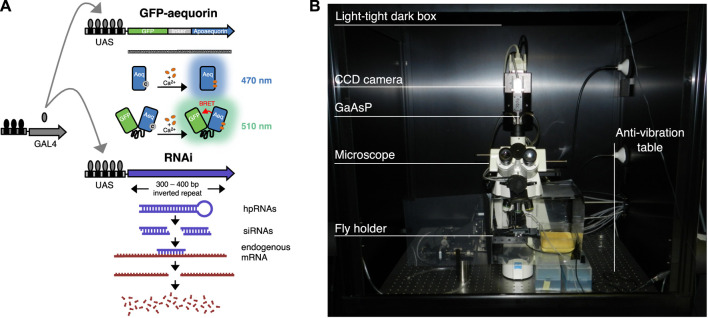
Genetic techniques and Ca^2+^ imaging setup. **(A)** GFP-aequorin responders activated by the driver GAL4 used in this study. On the top, schematic of GFP and aequorin fusion gene with upstream activation sequence (UAS). Models of blue light emission by aequorin (grey dot represents coelenterazine), and of green light emission by GFP-aequorin in response to high levels of Ca^2+^ (orange dots) (image adapted from Xiong et al., 2014). On the bottom, schematic of the RNAi technique in which a double-stranded RNA (i.e., hairpin RNA, hpRNA) is expressed under the control of UAS, as a complementary sequence to the gene of interest. The dsRNA is then processed by Dicer-2 into siRNA which leads to sequence-specific degradation of the mRNA related to the gene of interest (image adapted from https://stockcenter.vdrc.at/control/library_rnai). **(B)** Image of the setup used for *in vivo* Ca^2+^ brain imaging based on the bioluminescence technique.

Flies were maintained on standard medium at room temperature (24°C). Five to ten newly enclosed males and females per vial were kept for mating. A new version of the responder GFP-aequorin (G5A) placed downstream of 20 UAS repetitions (w^1118^;P{y^+t7.7^ w^+mC^ = 20xUAS-G5A}attP2) (a courtesy of Barret D. Pfeiffer, Janelia Research Campus, Ashburn, VA, United States) was used for crossings with the driver lines targeting tangential and columnar neurons (Pfeiffer et al., 2010; Jenett et al., 2012). To target the R2 neurons we used the w^1118^; P{y^+t7.7^ w^+mC^ = GMR20D01-GAL4}attP2 (BDSC #48889), while for the R5 neurons we used the w^1118^; P{y^+t7.7^ w^+mC^ = GMR72D06-GAL4}attP2 (BDSC #39769). The columnar E-PG neurons were targeted by using the w^1118^; P{y^+t7.7^ w^+mC^ = GMR70G12-GAL4}attP2 (BDSC #39552). Knockdown of dopamine 1-like receptors was performed by means of RNAi on Dop1R1 w^1118^; P{w^+mC^ = UAS-Dop1R1 RNAi}pKC43; (VDRC #107058/KK) and Dop1R2 w^1118^;P{w^+mC^ = UAS-Dop1R2 RNAi}pMF3; (VDRC #3392/GD) (Dietzl et al., 2007; Green et al., 2014). Therefore, *trans*-heterozygous lines bearing GAL4, 20xUAS-G5A and UAS-RNAi were also used (Figure 2A). Imaging experiments were performed on 4–5 days old mated females.

### Preparation of Flies

Flies ready to be tested were prepared for *in vivo* brain imaging experiments as described by Martin et al. (2007). Briefly, an offspring collected from food vials was ice-anesthetized and then, by gently grabbing its wings with a forceps, was positioned upside down on a plastic coverslip (22 × 22 × 0.157 mm) specifically designed to accommodate the upper half of its head inside a hole (BAH446900000-1 PK, Sigma-Aldrich, St. Louis, MO, United States). A drop of dental glue between the coverslip and the dorsal part of the fly’s thorax guaranteed the binding (Protemp 4^®^, 3M ESPE™, Seefeld, Germany, EU). The coverslip was previously prepared so as to have a hole of approximately 0.6 mm diameter drilled at the center by using an awl. Moreover, a thinner edge around this hole was made by using a cutting burr bit mounted on a rotary machine tool (Dremel 3,000, Dremel^®^, Mount Prospect, IL, United States). This latter operation was necessary in order to make room for the dissection and, at the same time, to leave the fly’s visual field intact in the lower half as much as possible. Once the fly was glued to the coverslip, its head was pushed through the hole in such a way that the antennae were below the horizontal plane of the coverslip and the rest of the head capsule above it. Then, the free space around the fly’s head was sealed using a special bio-compatible silicon (Kwik-Sil™, WPI, Sarasota, FL, United States). The entire preparation was fixed on an acrylic block with two small strips of adhesive tape whereby the fly was tethered but free to move its legs. Ringer’s solution with pH = 7.3 containing 130 mM NaCl, 5 mM KCl, 2 mM MgCl_2_, 2 mM CaCl_2_, 36 mM sucrose and 5 mM HEPES-NaOH (Martin et al., 2007), was dropped over the upper half of the head and a tiny window between the eyes and just above the antennae was opened in the head capsule using a micro knife (#10315-12, Fine Science Tools GmbH, Heidelberg, Germany, EU) to expose the brain. The underlying neural sheath was also gently removed with forceps (Dumont #5SF, Fine Science Tools GmbH, Heidelberg, Germany, EU) in order to improve the exposure of the outer brain surface. Extreme care was taken to avoid damaging the fly brain structures. The dissection procedure was performed under a fluorescence stereomicroscope (Leica MZ FLIII, Leica Microsystems GmbH, Wetzlar, DE, EU). After the dissection, the fly was free to recover for 2 h while its exposed brain was incubated in 100 μl of Ringer’s solution containing 25 μM native water-soluble coelenterazine (NanoLight^®^, Prolume Ltd., Pinetop, AZ, United States). Subsequent to the incubation, the preparation solution was replaced with 100 μl of fresh Ringer’s solution and the fly was ready to be imaged. In this condition, the fly was able to breathe via the tracheal system and could be maintained alive for more than 24 h. However, before starting the recording an air puff was delivered with a mouth aspirator onto the fly’s legs to stimulate a locomotor reflex. If the fly did not show any response, then it was not further considered for the experiment.

### *In Vivo* Brain Imaging

Bioluminescence signals (i.e., Ca^2+^-response) in tangential and columnar neurons were recorded using an intensified CCD camera with a cooled (at −20°C) GaAsP photocathode (Turbo-Z™, Stanford Photonics Inc., Palo Alto, CA, United States) fitted onto a direct microscope (Axioplan 2, Carl Zeiss GmbH, Jena, Germany, EU) (Figure 2B). The entire system was positioned on an anti-vibration table and housed inside a light-tight dark box (Science Wares Inc., Falmouth, MA, United States). Using a 20x water immersive objective lens (Zeiss N-Achroplan, N.A. 0.5) the spatial resolution was 480 × 360 μm (640 × 480 pixels), while using a 40x objective lens (Zeiss N-Achroplan, N.A. 0.75) it was 240 × 180 μm (1 pixels = 0.375 × 0.375 μm). To acquire and store data, each detected photon was assigned an x and y-coordinate and a time point (i.e., x, y, t). Photon acquisition was carried out at 120 frames s^−1^, providing 8.3 ms time resolution with an extremely low background signal. The Photon Imager software (Science Wares Inc., Falmouth, MA, United States) written in LabView 2010 (National Instruments™, Austin, TX, United States) was used for this purpose. Image recordings were obtained from 10 to 20 flies per each genotype. Differently to other fluorescence-based imaging approaches such as those which rely on GCaMP, bioluminescent GFP-aequorin does not require (and should not undergo) light excitation. This provides some advantages while avoiding some of the disadvantages associated with light excitable fluorophores. Specifically, the GFP-aequorin signal is not subject to disturbances from autofluorescence, phototoxicity and photobleaching. Consequently, it allows continuous real-time recordings, which in our case were performed with a 8.3 ms temporal resolution, during extended periods of time, in relatively deeply located structures (since it has no autofluorescence). However, since this approach does not rely on fluorophore excitation, it is not possible to measure ΔF/F, but rather we directly measure the number of emitted photons (Martin, 2008).

### Pharmacology

To stimulate the targeted neurons in flies we used either nicotine or picrotoxin. Depending on the putative nature of the presynaptic neurons in input to the circuit under investigation we applied these two drugs as stimulants for eliciting a Ca^2+^-response following activation of the excitatory nicotinic acetylcholine receptors, or blocking the GABA_A_ receptors, respectively. Nicotine (N3876, Sigma-Aldrich, St. Louis, MO, United States) was prepared as a 10 mM stock solution in H_2_O and diluted to 100 μM in Ringer’s solution as final concentration reached during the experiment (i.e., 1 μl application). Picrotoxin (P1675, Sigma-Aldrich, St. Louis, MO, United States) was prepared as a 25 mM stock solution in H_2_O and then dissolved in Ringer’s solution to 250 μM as final concentration reached during the experiment (i.e., 1 μl application). Dopamine (H8502, Sigma-Aldrich, St. Louis, MO, United States) was dissolved directly in Ringer’s solution prepared without sucrose at 1 mM and diluted to 100 μM as the result of a 10 μl application during the recording. Accordingly, we used Ringer’s solution instead of dopamine in the control samples. KCl application was also used at the end of each trial to evoke a strong Ca^2+^-response in order to check that the preparation was in good condition. KCl (P9333, Sigma-Aldrich, St. Louis, MO, United States) was prepared as a 3 M stock solution in H_2_O and diluted to 100 mM in the bathing solution during the experiment (i.e., 30 μl application). All drugs were applied to the bath solution using a pipette directly positioned on top of the brain.

### Data Processing

Pre-processing of imaging data was performed using the Photon Viewer (2.1) software (Science Wares Inc., Falmouth, MA, United States). Bioluminescence signals are presented as the total amount of emitted photons within a selected region of interest (ROI). Using GFP images of individual expression patterns, collected before the beginning of the experiments, we identified the ROIs and confirmed them by visual verification of the coverage of the subsequent response area. Different ROIs were manually drawn depending on the subclass of neurons investigated and the same shapes and sizes were kept constant among flies. To improve the signal-to-noise ratio, data were subjected to 1 s integration time (1 Hz) without applying any binning of pixels. The data frames were exported as “.csv” files and then imported into RStudio Team (2017) for data processing and subsequent statistical analysis. Duration, latency and total photons of the Ca^2+^-response were automatically computed for each ROI using a custom R script. We considered a response onset as a 10% increase in the number of photons s^−1^ with respect to a normalization performed on the basis of the maximum number of photons s^−1^ collected (i.e., the response peak). Moreover, to avoid the detection of unrelated activity such as the triggering of spots of increased photons s^−1^ on the part of rare cosmic rays, the response above the 10% threshold had to be sustained for at least 2 s. Accordingly, the end of the response was defined as the decrease in the photons s^−1^ below the 10% threshold. For the average profile, the alignment was performed on the response peak and the time window set to 200 s around the peak (i.e., 100 s before and 100 s after the peak).

### Statistical Approach

We adopted a Bayesian approach instead of a more “traditional” frequentist approach to analyze our data in order to accommodate the nature and structure of the data and to move forward with an approach that will probably contribute to improve the generality of the inferences, given the fact these data are not treatable (or poorly so) with the frequentist approach (Baker, 2016; Saravanan et al., 2020; DeBruine and Barr, 2021). A linear mixed effects (LME) model consists in a regression in which the parameters (i.e., the regression coefficients) are assigned to a probability model that is in turn estimated from the data (Gelman and Hill, 2007). In a LME model at least one predictor is categorical (e.g., experimental units such as the flies in our study) and defined by a set of discrete levels. The parameters associated to the specific levels of a predictor are called effects. They are called fixed effects when the set of levels is fixed and reproducible and they are called random effects when the levels constitute a random sample from the set of all possible levels (Bates, 2010). It is important to notice that the fixed effects parameters are actual parameters of the statistical model while the random effects are technically not parameters, but unobserved random variables (Bates, 2010). Mixed effects models are statistical models which include both fixed and random effects and the LME models are a specific class of these. They are also called hierarchical or multilevel models both because of the characteristics of the data structures they can handle and because of the hierarchy defined by the parameters of the model (Gelman and Hill, 2007). LME models allow the adjustment of estimates for repeated sampling and for imbalance in sampling (i.e., some groups with more individuals than others). Also, they take into account experimental variation (i.e., variation among flies or among other grouping variables) avoiding the harmful effects of averaging which very often reduce the statistical power of the analysis. For these reasons, LME models represent a reasonable way to strike a good balance between Type I error and power. We fitted the data with different LME models with the R package *lme4* (Bates et al., 2014). The models were then compared by using the Bayesian Information Criterion (BIC) in order to select the model with the greatest predictive power, given the experimental data against which it is tested (Schwarz, 1978). BIC is an index that measures the efficiency of the model in terms of data prediction. Furthermore, an approximation of the Bayes Factor (BF), calculated as

$$BF\;\approx e^{\left(\frac{\Delta BIC}{2}\right)}$$

where $\Delta BIC=BIC_{\left(2nd-best\right)}-\;BIC_{\left(1st-best\right)}$ , was computed to obtain a quantitative estimate of the degree of predictivity of the best model as compared to the second-best one (Raftery, 1995). The BF can be defined as the probability of a result under a specific hypothesis over another one. Pairwise post hoc comparisons adjusted with the Bonferroni correction were performed on the fixed effects of the best LME model with the R package *emmeans* (Lenth et al., 2022). Only the comparison referring to the EB was reported.

## Results

### Evoked Ca^2+^-Activity in the EB Neurons

To test if bath-applied drugs excited the EB neurons, we used an *in vivo* bioluminescence Ca^2+^ imaging technique as previously described (Martin et al., 2007; Pavot et al., 2015; Lark et al., 2016). By means of the GAL4-UAS binary system we expressed the bioluminescent molecule GFP-aequorin (G5A) in different EB neurons (Baubet et al., 2000; Martin et al., 2007). In order to narrow down the investigation to a few driver lines expressing GAL4 in selected EB neurons which were also deemed more likely to be modulated by dopamine, we screened the expression patterns of a number of GAL4 lines from the FlyLight database (Jenett et al., 2012). The prerequisites for a driver line to be included were the pattern of innervation at the level of the EB and concurrently that the fragment of DNA serving as transcriptional enhancer for GAL4 belonged to a dopamine receptor. Alternatively, the driver line had to show an expression pattern which highlighted innervation of the EB and which overlapped with the pattern of a GAL4 line, the transcriptional enhancer of which belonged to a dopamine receptor. In this way, three main GAL4 lines were selected for the experiments: two lines expressing in two different subclasses of R-neurons and one line expressing in the E-PG neurons.

**FIGURE 3:**
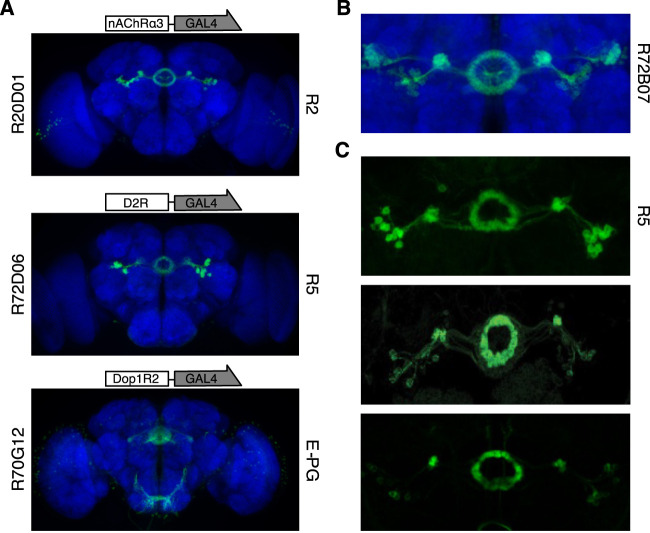
Driver lines selected and neurons targeted. **(A)** Images of the pattern expressed by the GAL4 lines selected from the Janelia FlyLight Project database (https://flweb.janelia.org/cgi-bin/flew.cgi) (Jenett et al., 2012). Starting from the top: the R20D01, with the promoter sequence corresponding to the putative enhancer sequence of the *nAChRα3* gene, targets the R2 neurons; in the middle the R72D06, with the promoter sequence of the *D2R* gene, targets the R5 neurons; and at the bottom the R70G12, with the promoter associated to *Dop1R2* gene, targets the E-PG neurons. **(B)** Expression pattern of R72B07-GAL4 (image from https://flweb.janelia.org/cgi-bin/flew.cgi) which has the promoter sequence associated to the *Dop1R1* gene. As can clearly be appreciated, its pattern is widely superimposable with that of R20D01, apart from the TuBu neurons. **(C)** Expression patterns of three different driver lines targeting the same subclass of neurons (i.e., R5). Starting from the top: image of the R58H05-GAL4 line expressing GFP (10xUAS-IVS-mCD8:GFP) which was considered by Omoto et al. (2018) to target the same neurons considered as R2 by Liu et al. (2016) and recently defined as R5 neurons (image from https://flweb.janelia.org/cgi-bin/flew.cgi); in the middle, pattern of the nv45-LexA:VP16 driver line expressing GFP (LexAop-mCD8:GFP) which was considered to target R3 neurons (image taken with permission from Kottler et al., 2017); at the bottom, image of the R69F08-GAL4 line (used by Liu et al., 2016) expressing GFP (10xUAS-IVS-mCD8:GFP) that was considered to target R2 neurons (image from https://flweb.janelia.org/cgi-bin/flew.cgi).

The first driver line targeting tangential neurons, the R20D01 (Figure 3A, top), is selective for the R2 neurons as defined by Omoto et al. (2018). This line actually has the transcriptional enhancer corresponding to a DNA fragment of the nicotinic acetylcholine receptor (nAChR) α3 subunit. Therefore, it is not directly a putative dopamine receptor-expressing line. However, it was selected because of the overlap in the expression pattern with another line targeting the R2 neurons (R72B07) which does have the putative enhancer fragment of the Dop1R1 receptor (Figure 3B). The latter driver line was not selected because its pattern includes TuBu neurons. Therefore, we surmised that the R2 neurons targeted by R20D01 express Dop1R1 besides nAChR and we pharmacologically stimulated them (R2 > G5A flies) using nicotine as the excitatory drug.

The second driver line targeting tangential neurons, the R72D06 (Figure 3A, middle), strongly labels what have recently been named R5 (Figure 3C, top) neurons (Omoto et al., 2018). These neurons seem to correspond quite closely to the neurons targeted by the nv45-LexA driver line which Kottler and coworkers have previously defined as R3 (Figure 3C, middle) neurons (Kottler et al., 2017). Furthermore, according to Omoto et al. (2018), R5 neurons well described those which in previous papers have been referred to as the R2 (Figure 3C, bottom) subclass (Liu et al., 2016; Donlea et al., 2018). The R72D06 driver line is characterized by a putative enhancer fragment corresponding to D2R. Since this line projects axons over the anterior surface of the EB, then into the canal and these then spread centrifugally, other neurons, probably R3w, may be targeted as well (Omoto et al., 2018). The expression pattern of another driver line (R70F01) (Jenett et al., 2012) with a putative enhancer fragment from the *resistant to dieldrin* (*Rdl*) gene, which encodes for the GABA_A_ receptor, targets a wide range of R-neurons (among them R5 and R3w) but substantially spares R2 neurons. For this reason, we pharmacologically disinhibited the R5 neurons (R5 > G5A flies) using picrotoxin, a non-competitive blocker of GABA_A_ receptor chloride channels.

Finally, the E-PG neurons were targeted by the driver line R70G12 which was characterized by a putative enhancer fragment from the Dop1R2 (Figure 3A, bottom). It is worth noting that, a recent cell-type-specific RNA sequencing (RNA-seq) of the E-PG neurons confirmed the expression of Dop1R2 and Dop1R1 (Turner-Evans et al., 2020). Moreover, since the columnar neurons have recently been proposed to be cholinergic (Franconville et al., 2018; Turner-Evans et al., 2020) and are characterized by an extensive recurrent networking to update the fly’s heading (Green et al., 2017; Turner-Evans et al., 2017), we stimulated the E-PG neurons (E-PG > G5A flies) using nicotine. Also in this case the expression of nAChR and metabotropic acetylcholine receptor (mAChR) in E-PG have been revealed in RNA-seq experiments (Turner-Evans et al., 2020).

**FIGURE 4:**
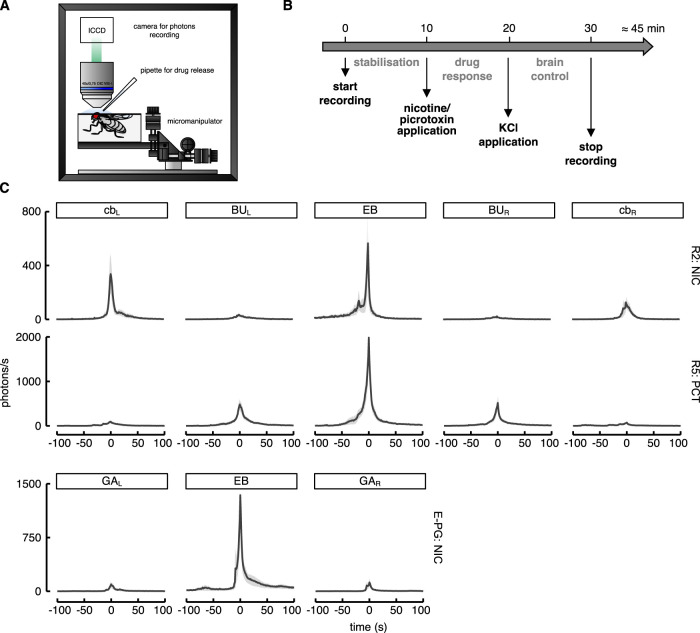
Experimental procedure and Ca^2+^-response to drugs application. **(A)** Cartoon of the fly preparation depicting the main components which are not drawn to scale. **(B)** Schematic drawing of the protocol used to stimulate the neurons. **(C)** Ca^2+^-response profiles to drug application in the selected ROIs (cb_L-R_: left and right cell bodies; BU_L-R_: left and right bulb; EB: ellipsoid body; GA_L-R_: gall region). Starting from the top: nicotine-evoked (NIC) activity of the R2 neurons (R20D01 driver) in the five ROIs drawn around cb, BU and EB (see also Supplementary Movie S1 for a representative brain response); in the middle picrotoxin-evoked (PCT) activity of the R5 neurons (R72D06 driver) in the five ROIs drawn as in the previous neurons (see also Supplementary Movie S2 for a representative brain response); at the bottom, nicotine-evoked (NIC) activity of the E-PG neurons (R70G12 driver) in the three ROIs drawn around the 2 GA regions and EB (see also Supplementary Movie S3 for a representative brain response).

To test whether the stimulant drugs were able to excite the cells under investigation, we applied the drugs during the Ca^2+^ imaging recordings (Figure 4A). After 10 min of baseline recording, the drug was applied to the preparation and the evoked response was recorded over a period of 10 min. At the end of this period, 10 μl of KCl was applied as a stimulus control to verify the integrity of the brain preparation (Figure 4B).

The results confirmed our expectations regarding the stimulating properties of the drugs used. In all genotypes, a Ca^2+^-response was evoked as a consequence of the application (Figure 4C) (see also Supplementary Movies S1–3 for a representative brain response for each driver line). Compared to the baseline activity, drug-evoked responses in the EB reached thousands of photons s^−1^ within a few seconds. Although a pharmacological approach makes it almost impossible to rule out indirect responses caused by the activation of neurons other than those specifically targeted, we believe that the bulk of these responses was likely determined by direct stimulation.

### Dopamine Modulation of Ca^2+^-Activity of the EB Neurons

**FIGURE 5:**
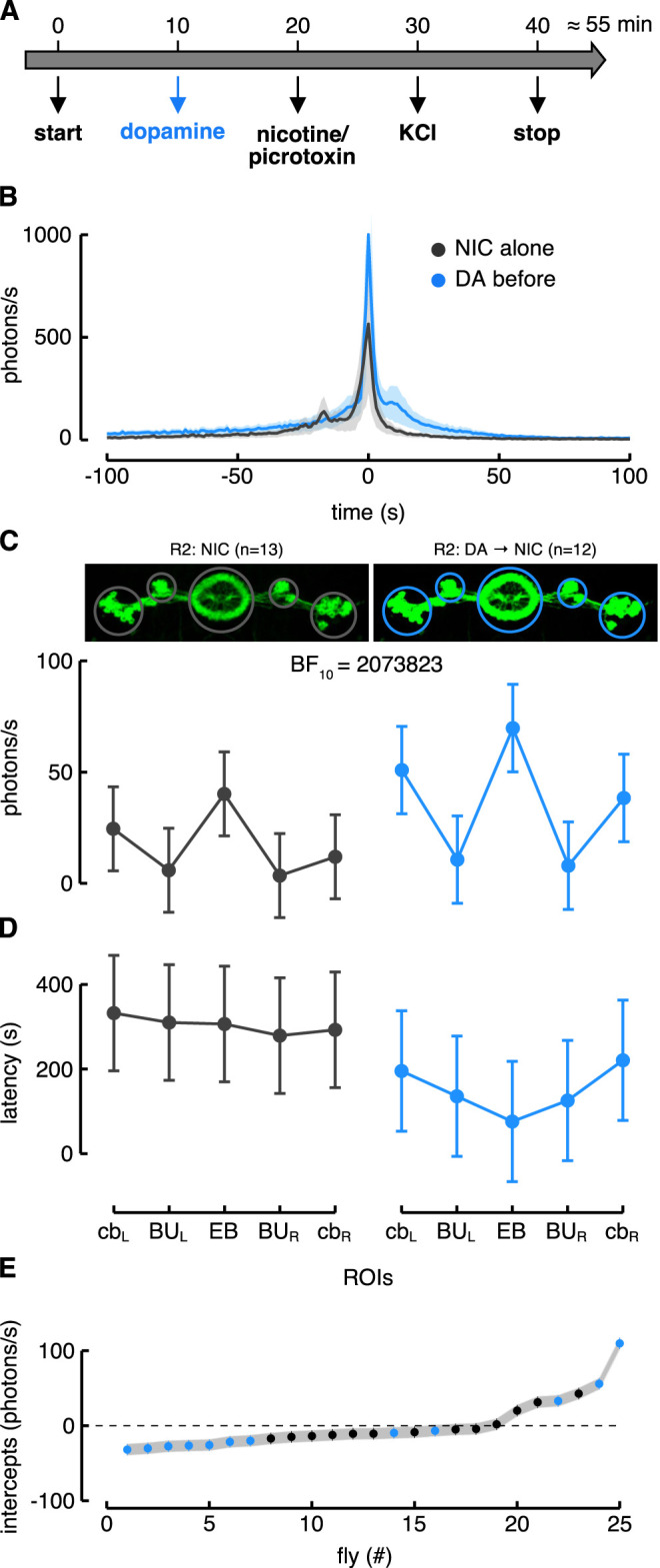
Dopamine modulation in R2 > G5A flies. **(A)** Image of the protocol used to modulate the neurons before the drug application (nicotine or picrotoxin). **(B)** Ca^2+^-response profiles of the R2 neurons in the EB ROI. In black is depicted the condition with the nicotine alone while in blue the condition with dopamine application before nicotine. **(C)** Estimated parameters of the Ca^2+^-response referred to the interaction between condition and ROIs (i.e., fixed effect). The dots represent the estimated values while the error bars correspond to the 97.5% confidence intervals (CI) computed with parametric bootstrap of 10,000 simulations. On the left is represented the Ca^2+^-response to nicotine (NIC) alone in the five ROIs of the R2 neurons (*n* = 13), while on the right is represented their Ca^2+^-response to nicotine after dopamine (DA) application (*n* = 12) (see also Supplementary Movie S4 for a representative brain response). **(D)** Estimated parameters of the response latency referred to the interaction between condition and ROIs (i.e., fixed effect) with corresponding 97.5% CI (computed as in Figure 5C). On the left is represented the latency response to nicotine alone in the five ROIs of the R2 neurons while on the right is represented their latency response to nicotine after dopamine application. **(E)** Plot of random effect referred to Ca^2+^-response (i.e., random fly intercept). Dots represent each fly (known as BLUPs, Best Linear Unbiased Predictions) while the horizontal lines crossing dots (i.e., error bars) correspond to the standard deviation (SD).

To test the hypothesis that these neurons could be subject to dopamine modulation, we modified the previous protocol by adding a dopamine application 10 min before the stimulation (Figure 5A). The R2 neurons showed an enhanced Ca^2+^-response to nicotine after dopamine application compared to the condition without dopamine (Figure 5B). However, to better understand the extent of the dopamine modulation, we fitted the data with different LME models considering several predictors: “condition” that distinguishes the responses with or without dopamine application; “ROIs” that refers to the different regions innervated by the driver line (e.g., cell body, EB and BU); and “time” that refers to the time window during which the responses were unfolded. For the R2 neurons we defined five ROIs encompassing all the EB, the two lateral superior BU and the two lateral groups of cell bodies (cb). We computed the BIC for the LME model comparisons and the best one took into account the interaction between “condition” and ROIs as fixed effects (Table 1).

**TABLE 1:** Models selection.

Model	Df	BIC
$Y_{ij}=\;\beta_{0}+\beta_{1}D_{1i}D_{2i}+\lambda_{i}+\varepsilon_{ij}$	12	312729.16
$Y_{ij}=\;\beta_{0}+\beta_{1}D_{1i}D_{2i}+\beta_{2}X_{1i}+\lambda_{i}+\varepsilon_{ij}$	13	312742.30
$Y_{ij}=\;\beta_{0}+\beta_{1}D_{1i}+\lambda_{i}+\varepsilon_{ij}$	7	312754.79
$Y_{ij}=\;\beta_{0}+\beta_{1}D_{1i}+\beta_{2}D_{2i}+\lambda_{i}+\varepsilon_{ij}$	8	312756.75
$Y_{ij}=\;\beta_{0}+\beta_{1}D_{1i}D_{2i}X_{1i}+\lambda_{i}+\varepsilon_{ij}$	22	312800.62
$Y_{ij}=\;\beta_{0}+\beta_{1}D_{2i}+\lambda_{i}+\varepsilon_{ij}$	4	313287.87

$Y_{ij}:$ Ca^2+^-response; $D_{1}:$ ROIs; $D_{2}:$ condition; $X_{1}:$ time; $\lambda_{i}:$ random effects; $\varepsilon_{ij}:$ error

This model showed a higher likelihood than the one that considered only the ROIs as a fixed effect (BF ≈ 2073823), meaning that the increase of the response following the dopamine application was not uniform among the five ROIs but some increased much more than others (Figures 5C,D) (see also Supplementary Movie S4 for a representative brain response). Specifically, while the response in the BU did not change so much, it increased in the perikarya and in the EB. The same model was also identified for the latency of the response that showed a reduction which was particularly evident in the EB (Figure 5E). These results corroborated the idea that the R2 neurons are excited by dopamine likely *via* Dop1R1 receptors. Noteworthy, after the dopamine application no increase in the Ca^2+^-response was ever recorded until nicotine was applied, meaning that dopamine did not have any effect on its own (data not shown).

**FIGURE 6:**
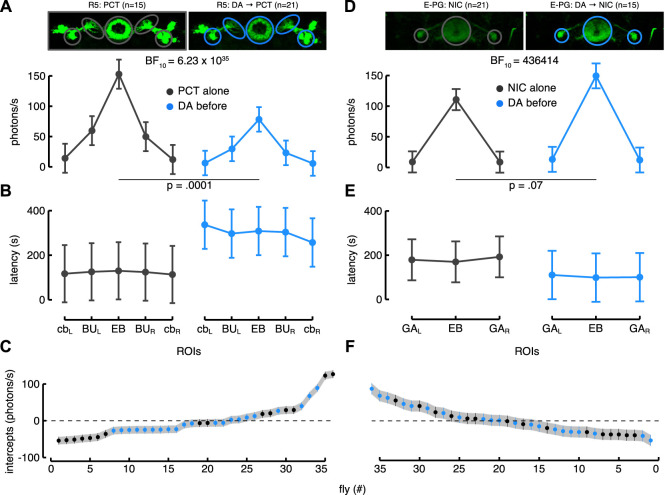
Dopamine modulation of R5 > G5A **(A–C)** and E-PG > G5A flies **(D–F)**. **(A)** Estimated parameters of the Ca^2+^-response referred to the interaction between condition and ROIs (i.e., fixed effect) with corresponding CI (computed as in Figure 5C). On the left is represented the Ca^2+^-response to picrotoxin (PCT) alone in the five ROIs of the R5 neurons (back, *n* = 15), while on the right is represented their Ca^2+^-response to picrotoxin after dopamine (DA) application (blue, *n* = 21) (see also Supplementary Movie S5 for a representative brain response). **(B)** Estimated parameters of the response latency referred to the interaction between condition and ROIs (computed as in Figure 5D). On the left is represented the latency response to picrotoxin alone in the five ROIs of the R5 neurons (black) while on the right is represented their latency response to picrotoxin after dopamine application (blue). **(C)** Plot of random effect referred to Ca^2+^-response of R5 neurons (as in Figure 5E). **(D)** Estimated parameters of the Ca^2+^-response referred to the interaction between condition and ROIs (as in Figure 5C). On the left is represented the Ca^2+^-response to nicotine (NIC) alone in the three ROIs of the E-PG neurons (black, *n* = 21), while on the right is represented their Ca^2+^-response to nicotine after dopamine (DA) application (blue, *n* = 15) (see also Supplementary Movie S6 for a representative brain response). **(E)** Estimated parameters of the response latency referred to the interaction between condition and ROIs (computed as in Figure 5D). On the left is represented the latency response to nicotine alone in the three ROIs of the E-PG neurons (black) while on the right is represented their latency response to picrotoxin after dopamine application (blue). **(F)** Plot of random effect referred to Ca^2+^-response of E-PG neurons (as in Figure 5E).

On the contrary, the other subclass of R-neurons, the R5, showed a clear reduction of the Ca^2+^-response to picrotoxin after dopamine application. As in the case of the R2 neurons, we tested and compared the same LME models. The ROIs for these neurons corresponded roughly to the ones of the R2, apart from the BU which were encompassed by larger ROIs because of the particular ramifications projecting to the EB. Again, the best model was the one that included the interaction between “condition” and ROIs. It showed a higher likelihood than the one with only ROIs as the fixed effect (BF ≈ 6.23 × 10^35^) but in this case the responses decreased as a consequence of dopamine application, especially in the BU and EB (Figures 6A,C) (see also Supplementary Movie S5 for a representative brain response). Still, the dopamine application increased the latency of the response as confirmed by the same model (Figure 6B). These results were consistent with the idea that R5 neurons express the inhibitory D2R receptors. We confirmed that dopamine modulates these neurons by inhibiting the response and increasing the response latency.

Dopamine application on the E-PG neurons moderately increased the Ca^2+^-response. Three ROIs were drawn, one encompassed the EB and two the GA regions. The deeper PB glomeruli innervated by these neurons and their perikarya were not considered because of difficulties in imaging the PB from above and to standardize a common ROI dimension for the cell bodies. Among the LME models tested, the best one confirmed the interaction between condition and ROIs as the fixed effect. This model showed a higher likelihood than the simpler model which did not consider the “condition” (BF ≈ 436414). Basically, the response did not change in the GA regions while it increased in the EB (Figures 6D,F) (see also Supplementary Movie S6 for a representative brain response) although, the response latency decreased quite uniformly for all three ROIs (Figure 6E). To sum up, the E-PG neurons were modulated by dopamine in terms of an increase in their activity.

### Knockdown of Dopamine Receptors in R2 Neurons

**FIGURE 7:**
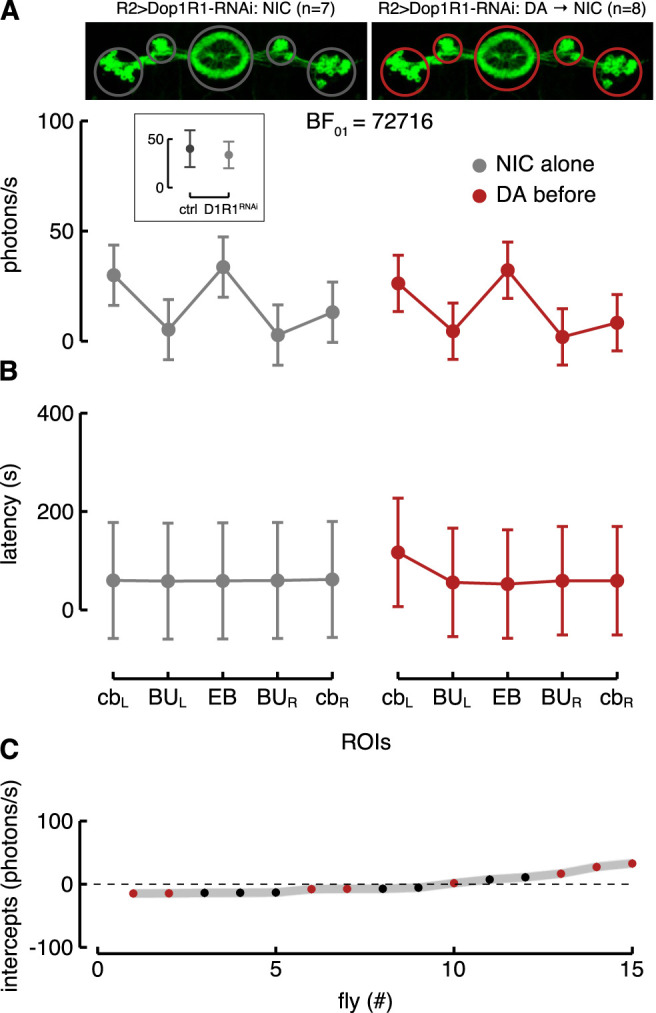
Knockdown of Dop1R1 in R2>G5A flies. **(A)** Estimated parameters of the Ca^2+^-response referred to the interaction between condition and ROIs with corresponding CI (computed as in Figure 5C). The inset represents the comparison of the estimated interaction parameters (i.e., between condition and ROIs) referred to EB in the R2 normal (ctrl) and R2 >Dop1R1-RNAi (D1R1^RNAi^) flies in response to nicotine. On the left is represented the Ca^2+^-response to nicotine (NIC) alone in the five ROIs of the R2 neurons with knockdown of Dop1R1 (R2 > Dop1R1-RNAi; grey, *n* = 7), while on the right is represented their Ca^2+^-response to nicotine after dopamine (DA) application (red, *n* = 8). **(B)** Estimated parameters of the response latency referred to the interaction between condition and ROIs (computed as in Figure 5D). On the left is represented the latency response to nicotine alone in the five ROIs of the R2 neurons with knockdown of Dop1R1 (grey) while on the right is represented their latency response to nicotine after dopamine application (red). **(C)** Plot of random effect referred to Ca^2+^-response (as in Figure 5E).

Since the targeted R2 neurons expressed GAL4 under the control of an enhancer fragment which was not consistent with a dopamine receptor, we directly tested whether the Dop1R1 (likely expressed by these neurons) affected their nicotine-evoked response and if dopamine increased their excitability *via* those specific receptors. For this purpose, we knocked down the Dop1R1 in R2 neurons by using RNAi (R2 > G5A + Dop1R1^RNAi^). Flies with downregulation of Dop1R1 in the R2 neurons showed a Ca^2+^-response to nicotine similar to the one of control flies (Figure 7A, inset) as shown by the best model considering only ROIs as fixed effect (BF ≈ 23174, against the model with ROIs and condition as fixed effects). Interestingly, dopamine application prior to nicotine did not increase the excitability as seen in the control flies (Figures 7A,C). By comparing different LME models, the best one considered only ROIs as the fixed effect (BF ≈ 72716), meaning that the Ca^2+^-response to nicotine was not increased as a consequence of dopamine application in R2 > G5A + Dop1R1^RNAi^ flies. In other words, the dopamine effect on the R2 neurons (shown in Figures 5B,C) is likely due to the Dop1R1 in the absence of which the increase in excitability is lost. On the contrary, a marginal effect due to dopamine was detected by the best model with respect to the response latency, which remained only slightly more likely than the one which did not employ “condition” as a predictor (Figure 7B). An interesting point in this regard is the fact that independently from dopamine, overall, the response latencies were reduced in R2 > G5A + Dop1R1^RNAi^ compared to R2 > G5A flies (i.e., in Figure 7B the average latency is only 60 s; while in Figure 5E it is 304 s).

### Opposite Modulation Operated by Dop1R1 and Dop1R2 in E-PG Neurons

**FIGURE 8:**
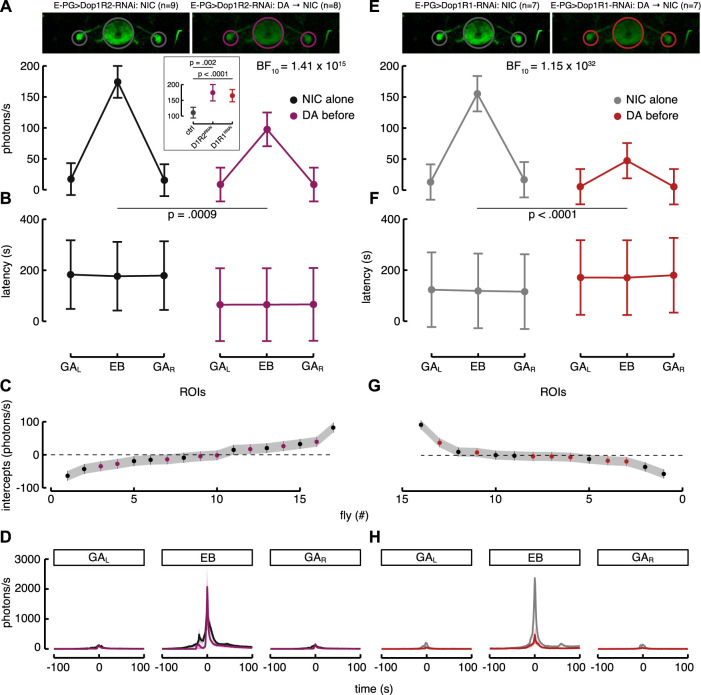
Knockdown of Dop1R2 **(A–D)** and Dop1R1 (E–H) in E-PG > G5A flies. **(A)** Estimated parameters of the Ca^2+^-response referred to the interaction between condition and ROIs (as in Figure 5C). The inset at the center represents the comparisons of the estimated interaction parameters (i.e., between condition and ROIs) referred to EB in the E-PG normal (ctrl), E-PG > Dop1R2-RNAi (D1R2^RNAi^) and E-PG > Dop1R1-RNAi (D1R1^RNAi^) flies in response to nicotine (post hoc comparisons adjusted with Bonferroni correction). On the left of the inset is represented the Ca^2+^-response to nicotine (NIC) alone in the three ROIs of the E-PG neurons with knockdown of Dop1R2 (E-PG > Dop1R2-RNAi; black, *n* = 9), while on the right is represented their Ca^2+^-response to nicotine after dopamine (DA) application (magenta, *n* = 8). **(B)** Estimated parameters of the response latency referred to the interaction between condition and ROIs in E-PG > Dop1R2-RNAi flies (computed as in Figure 5D). On the left is represented the latency response to nicotine alone in the three ROIs of the E-PG neurons with knockdown of Dop1R2 (black) while on the right is represented their latency response to nicotine after dopamine application (magenta). **(C)** Plot of random effect referred to Ca^2+^-response of E-PG neurons with Dop1R2 knockdown (as in Figure 5E). **(D)** Ca^2+^-response profiles to nicotine of the E-PG neurons with Dop1R2 knockdown in the three ROIs with (magenta) or without (black) dopamine application before. Starting from the left: left GA region, EB and right GA region. **(E)** Estimated parameters of the Ca^2+^-response in E-PG neurons with knockdown of Dop1R1 referred to the interaction between condition and ROIs. On the left the condition with nicotine (NIC) alone (E-PG > Dop1R1-RNAi; grey, *n* = 7) while on the right the condition with dopamine (DA) application before nicotine (red, *n* = 7). **(F)** Estimated parameters of the response latency in E-PG neurons with knockdown of Dop1R1 referred to the interaction between condition and ROIs (computed as in Figure 5D). On the left the condition with nicotine alone (grey) while on the right the condition with dopamine application before nicotine (red). **(G)** Plot of random effect referred to Ca^2+^-response of E-PG neurons with Dop1R1 knockdown. **(H)** Ca^2+^-response profiles to nicotine of the E-PG neurons with Dop1R1 knockdown in the three ROIs defined as in G with (red) or without dopamine application before (grey).

The same approach was used to clarify the neural response of the E-PG neurons. Inasmuch as the driver we used has an enhancer fragment relating to Dop1R2 and previous electrophysiological data have shown an inhibitory role carried out by Dop1R2 in sleep-promoting neurons (Pimentel et al., 2016), we expected a downward modulation. Surprisingly, our results showed an enhancement of the Ca^2+^-response after dopamine application (Figure 6D). Thus, to better understand which were the dopamine receptors involved, we knocked down the Dop1R2 with RNAi (E-PG > G5A + Dop1R2^RNAi^). The downregulation of the Dop1R2 in the E-PG neurons determined an increase in the Ca^2+^-response to nicotine compared to the non-compromised E-PG (Figure 8A, inset). The best model confirmed the interaction between “condition” and ROIs, meaning that the reduction of the Dop1R2 (as well as Dop1R1) took the E-PG neurons to a higher level of excitability within the EB (BF ≈ 2.12 × 10^23^). These data suggest that Dop1R2 are inhibitory receptors acting to maintain a low EB activity. Nevertheless, dopamine application before nicotine determined a reduction of the Ca^2+^-response in E-PG > G5A + Dop1R2^RNAi^ flies compared to nicotine application alone (BF ≈ 1.41 × 10^15^) as shown by the best model (Figures 8A,C). Moreover, the response latency to nicotine showed a reduction when dopamine was previously applied (Figure 8B). Noteworthy, this overall response reduction was fundamentally due to its brief duration which was actually characterized by a higher peak (Figure 8D). In other words, dopamine application in E-PG > G5A + Dop1R2^RNAi^ flies appeared to produce a greater (i.e., higher amplitude) nicotine-evoked response which was however very localized in time. These results suggest that other parallel actors were responsible for the complex response due to dopamine. The indirect dopamine increase in R2 neurons could be one of them. Specifically, increasing the GABAergic tone of R2 neurons may result in overall inhibition of the E-PG neurons. Alternatively, another subtype of dopamine receptors such as the Dop1R1 may be implicated in this complex response. To test whether and how this receptor was involved in the dopamine modulation of the E-PG neurons, we performed the same experiments, by knocking down Dop1R1 (E-PG > G5A + Dop1R1^RNAi^). Surprisingly, as seen for E-PG > G5A + Dop1R2^RNAi^ flies, the knock down of the Dop1R1 increased the E-PG response to nicotine (Figure 8A, inset). However, dopamine application strongly decreased (BF ≈ 1.15 × 10^32^) the Ca^2+^-response to nicotine in E-PG > G5A + Dop1R1^RNAi^ individuals (Figures 8E,G). A slight increase in the response latency was also observed after dopamine application (Figure 8F). Overall, these data converge towards a smaller, shorter and delayed nicotine-evoked response when dopamine was previously applied in E-PG > G5A + Dop1R1^RNAi^ flies (Figure 8H). Therefore, it is likely that in these flies an imbalance towards the Dop1R2 expressed by the E-PG neurons was the cause of their response to dopamine.

## Discussion

In primates action selection is mainly dependent on a group of subcortical nuclei called the basal ganglia, the functions of which are critically dependent on dopamine (DeLong, 1990; Redgrave et al., 1999). Dysfunction of these nuclei may result in several pathological conditions related to motor control such as Parkinson’s or Huntington’s diseases (Redgrave et al., 2010; Nelson and Kreitzer, 2014). Two main pathways, which consist of striatal projections to the basal ganglia output nuclei, control the selection process: the direct and the indirect pathway. These two pathways act together to perform action selection by disinhibiting a selected motor program while inhibiting all the other competing ones (Grillner et al., 2005).

The direct pathway is characterized by GABAergic striatal projection neurons—expressing excitatory dopamine D1 receptors—which inhibit, upon stimulation, the tonically active GABAergic output neurons that keep the brainstem motor centers inhibited (Grillner and Robertson, 2016). On the other hand, the indirect pathway is characterized by GABAergic striatal projection neurons—expressing inhibitory dopamine D2 receptors—which inhibit the GABAergic neurons of the globus pallidus *externa* that in turn inhibit the GABAergic output neurons (Grillner and Robertson, 2016).

The same architecture has also been identified in an organism belonging to the phylogenetically oldest group of vertebrates, the lamprey, which diverged from the evolutionary line leading to primates some 560 million years ago (Ericsson et al., 2011; Stephenson-Jones et al., 2012). This suggests that the mammalian basal ganglia evolved through a functional replication of these circuits rather than by means of a sequential adaptation of this ancestral architecture (Stephenson-Jones et al., 2011). Moreover, the striking conservation between such evolutionary distant organisms has also been demonstrated with respect to dopamine modulation, suggesting a truly common blueprint for the evolution of the basal ganglia (Ericsson et al., 2013; Stephenson-Jones et al., 2013).

**FIGURE 9:**
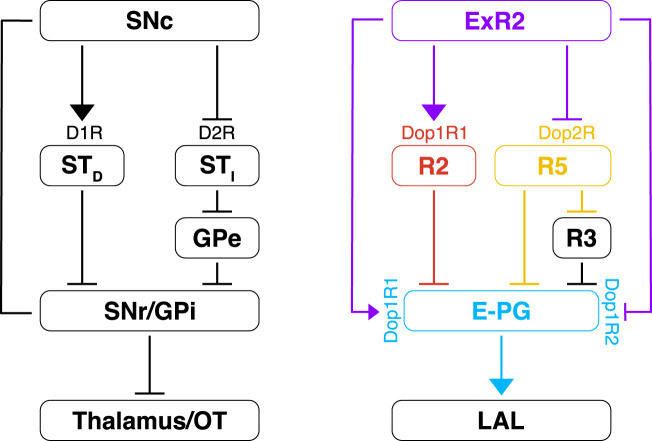
Dopamine modulation of the EB and comparison with the vertebrate basal ganglia. On the left is depicted the dopamine circuit involved in the vertebrate basal ganglia. The dopamine release from the substantia nigra *pars compacta* (SNc) modulates the direct and the indirect pathway. The striatal neurons (ST_D_) of the direct pathway express the D1R (excitatory) and they inhibit the GABAergic neurons of the substantia nigra *pars reticulata* (SNr) and globus pallidus *interna* (GPi). In the indirect pathway, the striatal neurons express the D2R (inhibitory) and they inhibit the GABAergic neurons of the globus pallidus *externa* (GPe) which, in turn, inhibit the GPi. Both these pathways converge back to the thalamus (with efference copies) and to motor command regions like the optic tectum (OT). On the right is proposed a simple model about the dopamine modulation of the CX. As for the vertebrate basal ganglia, dopamine might excite (*via* Dop1R1) and inhibit (*via* Dop2R) specific R-neurons, R2 and R5, respectively, which synapse directly and/or indirectly to the E-PG neurons. A combination of excitatory (Dop1R1) and inhibitory (Dop1R2) dopamine receptors would be expressed by the E-PG neurons. The overall computation could serve the selection of goal-directed behaviors that is subsequently passed to the LAL for motor control. T-shaped bars mean inhibitory connections while arrows mean excitatory connections.

Extending the comparison to invertebrates, fly dopaminergic PPM3 neurons have been compared to the mammalian *pars compacta* nucleus of the *substantia nigra* (Strausfeld and Hirth, 2013a). This similarity has been considered to be due to homology. Nonetheless, the speculative extension of this claim remains quite large and still under discussion (Farries, 2013). Indeed, it might be due to convergent or parallel evolution rather than to homology. In any case, regardless of these intriguing evolutionary issues, the main correspondence between CX and basal ganglia—that is the presence of pathways differently modulated by dopamine (Figure 9) has remained elusive up to now.

Kong et al. (2010) showed that PPM3 stimulation increased locomotor activity levels and that the Dop1R1 receptors in the R2 neurons are essential for ethanol-induced hyperactivity. Accordingly, a reduction of Dop1R1 in R2 neurons resulted in decreased ethanol-induced hyperactivity. This resembles the vertebrate direct pathway in which the D1 receptors act by increasing the response of the striatal projection neurons. Here, through a neurophysiological approach we showed the existence of a similar mechanism in which dopamine release on the R2 neurons modulates their response by increasing the Ca^2+^-activity *via* Dop1R1. Noteworthy, R2 neurons have been shown to respond to visual properties and to increase the walking probability (Seelig and Jayaraman, 2013; Robie et al., 2017).

On the contrary, a homologous indirect pathway in flies has never been proposed. Although Draper et al. (2007) showed that D2R plays a critical role in modulating locomotion and that reduced expression of this receptor resulted in decreased locomotor activity, no specific neuroanatomical structure or neuronal pathways have been identified at the core of this behavior. Administration of the synthetic D2 agonist bromocriptine, a well-established human anti-Parkinson drug, was also able to restore the deficit determined by the D2R knockdown (Draper et al., 2007). Strikingly, we identified a similar D2-based modulatory pathway involving R5 neurons. Our data suggest that dopamine release on the R5 neurons would act by inhibiting their activity very likely via D2R. A recent connectome analysis of the CX has highlighted that the PPM3-EB neurons (also known as ExR2) send outputs mainly to the superior BU where R2, R3w and R5 receive inputs, while a combination of input-output connections innervate the EB sending inputs to the E-PG neurons (Hulse et al., 2021). In this respect, Liang et al. (2019) have recently shown how the circadian M- and E-cells regulate the dawn and dusk peaks in locomotor activity by activating R5 neurons through the PPM3-EB. Furthermore, they showed an impaired locomotor activity in flies with Dop1R2 and D2R knock down in R-neurons (Liang et al., 2019).

Previous reports have shown that the vast majority of the R-neurons are GABAergic (Hanesch et al., 1989; Zhang et al., 2013; Fisher et al., 2019; Kim et al., 2019) and particularly the R2 neurons (Isaacman-Beck et al., 2020). By using the EB1 driver line which targets what has previously been referred to as R2/R4_m_, 94% of them were estimated to be GABAergic (Kottler et al., 2017). Moreover, an inter-R-neurons connectivity between R5 and R3 (ER3a specifically) has recently been shown in an electron microscopy (EM) reconstruction of the CX. Considering this connectivity and the fact that mainly the R5 neurons synapse onto the R3 neurons, these last neurons may be inhibited by the former ones via GABA. A structural connectivity between R2 and E-PG neurons has been shown in GRASP, trans-Tango and in EM reconstructions as well (Omoto et al., 2018; Kottler et al., 2019; Hulse et al., 2021). The R2 neurons would inhibit the E-PG neurons via GABA_A_ receptor. On this matter, the R70F05 line (Jenett et al., 2012) has the putative enhancer fragment corresponding to *Rdl* and it targets the E-PG neurons. A corroboration of the fact that E-PG neurons express Rdl receptors comes from RNA-seq experiments (Turner-Evans et al., 2020). In this sense, R2 neurons, after being activated by the visual system *via* nAChR, would globally inhibit the E-PG neurons but not uniformly so as to push the bump of activity towards the E-PG neurons that are the least inhibited (Fisher et al., 2019; Kim et al., 2019). In other words, since the inhibition of the R2 neurons towards the E-PG neurons is not equally distributed around the toroid, the excitatory drive arriving to the E-PG neurons would activate the less inhibited ones. In parallel, the E-PG neurons would combine the visual information with proprioceptive inputs arriving onto the PB and, in turn, sent to the EB through the P-EN neurons which update the fly’s heading (Green and Maimon, 2018). Similarly, the P-EG neurons, which may receive motor efference copies or proprioceptive inputs in the PB from other neurons, inhibit the E-PG neurons by passing through a class of GABAergic interneurons defined GB-Eo (GA-BU-outer EB) which receive inputs in the GA region and send outputs in the EB (Franconville et al., 2018). An important bottleneck of this complex circuit is represented by the Δ7 neurons which receive inputs and relay them within the PB (Wolff et al., 2015). These glutamatergic neurons receive activating inputs from the E-PG neurons and their activation stabilizes the heading representation by inhibiting—likely through GluC1α channels—only the subset of E-PG within one column in the PB (Franconville et al., 2018; Turner-Evans et al., 2020). This means that the bump of activity in Δ7 neurons has exactly the opposite angular orientation as the E-PG bump.

In our study, the E-PG neurons have shown to be modulated by dopamine which acts on two different subtypes of dopamine 1-like receptors, Dop1R1 and Dop1R2. Although an indirect dopamine modulation of the E-PG neurons remains a possible confounding effect, our data suggest that the Dop1R1 may work by shaping a sharp response in the E-PG neurons. Whereas, the Dop1R2 may work by decreasing the overall response. A dopamine modulation of E-PG neurons via Dop1R1 has also been demonstrated in behavioral experiments (Kottler et al., 2019). The functional role played by the dopamine in the EB might be to improve the signal-to-noise ratio in order to increase the robustness of the activity with respect to interfering input as shown in a network model of the human prefrontal cortex (Durstewitz et al., 2000). In the context of goal-directed behaviors, in order to accomplish the action selected, flies committed to reach a specific visual target might be induced to ignore distracting visual stimuli, through dopamine modulation of the EB (Frighetto et al., 2019). In patients with Parkinson’s disease, dopaminergic medication has shown to reduce interference effects on the motor program elicited by a target object when distracter objects evoke competitive motor programs (Castiello et al., 2000). The dopaminergic neurons would gate and invigorate movements that are planned somewhere else, likely in the cortex (Klaus et al., 2019). In primates, basal ganglia have shown to confirm the commitment to the decision by providing a signal that modulates the gain of how sensory evidence influences the cortex (Thura and Cisek, 2017). Therefore, even though the basal ganglia may not be the place where a specific motor plan is selected, they would still be needed for initiating it and for committing to it.

In flies, it is difficult to discretely localize the locus in which action selection may be implemented. However, the structure in which the vast majority of different inputs converges—including the ones from the mushroom-bodies (Zhang et al., 2013)—and that recursively interacts with them, is the EB. A recurrent networking among different sensory inputs at the basis of the integration would produce a sort of attentional focus as a byproduct, and this would serve the selection of action (Krauzlis et al., 2014; de Bivort and van Swinderen, 2016). Future work combining Ca^2+^ imaging and optogenetic manipulation in behaving flies, will probably disentangle this complex and fascinating issue.

## Data Availability

The original contributions presented in the study are included in the article/Supplementary Material, further inquiries can be directed to the corresponding author.
